# Endoplasmic Reticulum Stress in Neurodegenerative Diseases

**DOI:** 10.3390/jdad1020006

**Published:** 2024-10-14

**Authors:** Syed-Abdul-Moiz Hasan, Antonisamy William James, Farzeen M. Fazili, Samiha Tarabishi, Namir M. Sheikh, Zahoor A. Shah

**Affiliations:** 1 Department of Pharmacology and Experimental Therapeutics, College of Pharmacy and Pharmaceutical Sciences, The University of Toledo, Toledo, OH 43614, USA; 2 Department of Medicinal and Biological Chemistry, College of Pharmacy and Pharmaceutical Sciences, The University of Toledo, Toledo, OH 43614, USA

**Keywords:** ER stress, neurodegenerative diseases, unfolded protein response, neuroinflammation, neuronal death, Alzheimer’s disease

## Abstract

Endoplasmic reticulum (ER) stress is a detrimental cellular phenomenon in the cells and is activated by the accumulation of unfolded or misfolded proteins in the ER. The unfolded protein accumulation activates the unfolded protein response (UPR), an adaptive mechanism designed to mitigate cellular stress by enhancing the ER’s protein-folding capacity and protecting cells from apoptotic stimuli in neuroinflammation and neurodegenerative diseases. However, chronic ER stress and prolonged activation of the UPR can have adverse effects, including the activation of pro-apoptotic and inflammatory signaling pathways, which contribute to the development and progression of neurodegenerative disorders. Neurodegenerative diseases are complex and devastating conditions with underlying pathogenesis that are not fully understood. Genetic mutations leading to the accumulation of misfolded or phosphorylated tau proteins and amyloid-beta in the ER can induce ER stress, resulting in neuroinflammation and neuronal death. Several studies have reported the involvement of increased ER stress and UPR signaling proteins in the pathogenesis and progression of neurodegenerative diseases. Thus, inhibiting ER stress and neuroinflammation and targeting their associated signaling pathways represent a significant area of research interest. This review discusses the critical signaling molecules involved in ER stress, their mechanisms in the progression of neurodegenerative diseases, and the latest developments in the available ER stress inhibitors. Despite the extensive development of ER stress inhibitors over the years, only a limited number have been approved as pharmaceutical drugs. There remains a critical need for effective ER stress inhibitors to provide efficient treatments for neurodegenerative diseases, including Alzheimer’s disease.

## 1. Introduction

Neurodegenerative disorders (NDs) are a broad category of neurological illnesses characterized by the gradual degradation of neurons, resulting in the loss of motor function, cognitive deterioration, and other neurological symptoms. NDs are the primary cause of physical and cognitive disability, affecting approximately 15% of the total global population. These diseases have only become increasingly common over the past few decades. Parkinson’s disease (PD), Alzheimer’s disease (AD), Huntington’s disease (HD), and amyotrophic lateral sclerosis (ALS) are among the numerous disorders that fall under the broad category of NDs, but are marked by their distinct clinical appearances, afflicted brain areas, and underlying pathogenic mechanisms. In 2023, approximately 6.7 million Americans aged 65 and older had AD [1].

NDs are complex, arising from various factors, encompassing genetics, environments, and lifestyles. NDs are characterized by several variables, including oxidative stress, neuroinflammation, mitochondrial dysfunction, and pathological mechanisms such as protein aggregation and misfolding. Among these, endoplasmic reticulum (ER) stress has gained significant attention as a critical contributor to neurodegeneration [1].

The ER is a crucial organelle in eukaryotic cells, primarily responsible for synthesizing, folding, and modifying proteins. Proper protein folding is essential for all cellular functions, and the ER is equipped with various methods to ensure this process occurs correctly. The ER can be classified into two distinct forms: rough ER, which is embedded with membrane-bound ribosomes responsible for the production, folding, and control of the proteins, and smooth ER, which is associated with lipid and enzyme manufacture, as well as a detoxification function, and is not embedded with ribosomes [2].

During homeostasis, proteins synthesized in the ER are correctly folded with the assistance of various molecules. However, disturbances in ER homeostasis, caused by factors such as physiologic stresses, environmental toxins, or pathological stresses, lead to the accumulation of misfolded or unfolded proteins, resulting in an imbalance between the demand for protein folding and the capacity of the ER to perform protein folding, thereby causing ER stress. This accumulation triggers an evolved cellular stress response known as the unfolded protein response (UPR). The UPR aims to restore ER function by halting protein translation, upregulating chaperone proteins, and enhancing the degradation of misfolded proteins through the ER-associated degradation (ERAD) pathway, ultimately promoting apoptosis [3]. While the UPR initially serves a protective role, the chronic activation of this response can result in detrimental outcomes. Prolonged ER stress, often caused by an increase in the protein level before the restoration of homeostasis, can overwhelm the capacity of the UPR, resulting in the sustained promotion of inflammation and cell death [3].

This sustained activation has a distinct effect on the neurological system, as it is particularly detrimental in neurons, which have limited regenerative capacity and are, therefore, more susceptible to apoptosis. Chronic ER stress can also exacerbate NDs through protein aggregation. Persistent ER stress hinders the ER’s ability to appropriately synthesize proteins, resulting in the accumulation of misfolded and unfolded proteins, and it also overwhelms the ERAD pathway, enabling the misfolded proteins to go undetected. Therefore, chronic ER stress is implicated in the pathogenesis of various neurodegenerative diseases, as the accumulation and aggregation of misfolded proteins are central to the pathology of many neurodegenerative diseases, including AD, HD, PD, and ALS, since these disorders are often aggravated by protein aggregation in neurons.

Further, ER stress inhibitors can modulate and regulate inflammation, so their clinical use could be advantageous in treating neuroinflammatory diseases such as AD or PD. ER stress inhibitors are a class of compounds designed to modulate the UPR and ERAD, thereby restoring homeostasis and preventing neuronal damage and apoptosis. These compounds demonstrate neuroprotective potential through various mechanisms, including enhancing protein folding capacity, reducing oxidative stress, promoting autophagy, and facilitating the clearance of misfolded proteins. Through these mechanisms, ER stress inhibitors can mitigate the adverse effects of chronic ER stress and potentially alter the progression of neurodegenerative diseases.

## 2. ER Stress

ER stress is the disruption of the normal functioning of the ER that occurs because of the excessive misfolding of proteins in the ER. Misfolded proteins produced in the ER move to the cytosol and are degraded by the ubiquitin–proteasome pathway. If misfolded proteins accumulate in the cytosol, ER stress and the UPR can occur. The UPR is regulated by three ER sensors: inositol-requiring enzyme 1 (IRE1), RNA-activated protein kinase R (PKR), and activating transcription factor 6 (ATF6) [3].

**Figure 1.**
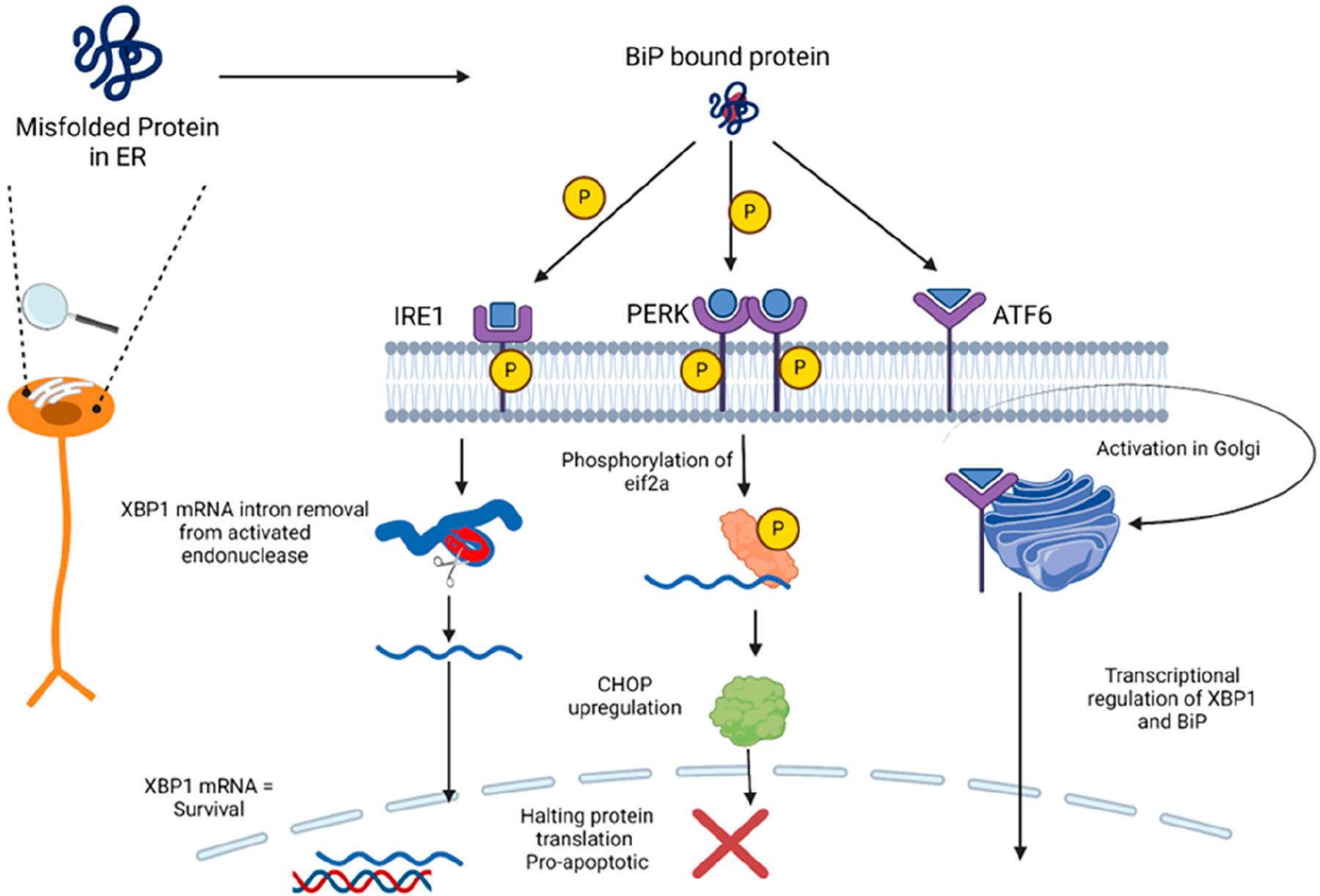
ER stress mechanisms for unfolded protein response (UPR). Created in BioRender. Hasan, S. (2024) BioRender.com/r17z787.

If ER stress in the brain becomes too severe, neurons start losing the ability to initiate autophagy, leading to cell death and conditions such as PD and AD. Therefore, ER stress inhibitors might mitigate such disease conditions [4]. These transduction pathways are known as inositol-requiring protein 1 (IRE1), protein kinase RNA-like ER kinase (PERK), and activating transcription factor 6 (ATF6) [5]. These transduction pathways are essential for regulating proteostasis. Under the conditions of neuronal homeostasis, these proteins are bound to glucose-regulated protein 78 (GRP78)/immunoglobulin heavy-chain-binding protein (BiP), an ER chaperone. BiP dissociates from IRE1, PERK, and ATF6 during ischemic stroke and initiates the UPR [6]. PERK is a type 1 transmembrane protein that increases phosphorylation activity during ER stress. PERK phosphorylates eukaryotic initiation factor-2α (eIF2α) at serine residue 51, which prevents the translation of messenger RNA (mRNA) into protein [7], thereby reducing the number of misfolded proteins. ATF6 is a type 1 transmembrane protein that, upon dissociation from BiP, is transported into the Golgi apparatus. Thereafter, ATF6 is cleaved and converted into a smaller, active form of ATF6. ATF6 is then transported to the nucleus and acts as a transcription factor, upregulating the transcription of pro-UPR proteins such as BiP. IRE1, under ER stress conditions, is dissociated from BiP. BiP phosphorylates IRE1, thereby activating its endonuclease activity, allowing for the removal of an intron in the gene encoding X-box-binding protein 1 (XBP1) mRNA. The protein encoded from this processed mRNA aggregates during the UPR and acts as a transcription factor by translocating to the nucleus, binding directly to DNA, and upregulating genes to enhance the capability of the cell to mitigate the effects of misfolded proteins [8]. Figure 1 shows the general pathway for each of these ER stress responses.

ER stress, in addition to increased apoptotic signaling, primarily occurs in the hippocampus compared to other regions in the brain [9]. ER stress affects several types of cells. ER stress is known to have implications in several neurodegenerative diseases associated with neural apoptosis, including AD and PD [10]. ER stress causes astrocytes to produce several inflammatory molecules. Additionally, astrocytes express IRE1, PERK, and ATF6, indicating its ability to initiate the UPR. ER stress increases the concentration of neuroprotective microglia, but prolonged stress causes inflammatory microglia to be of higher concentration [11]. ER stress allows for the survival of fully myelinated oligodendrocytes but causes the death of oligodendrocytes that are actively being myelinated [2].

## 3. Neurodegenerative Diseases Involving ER Stress

Endoplasmic reticulum stress plays a vital role in the etiology of different neurodegenerative and neuroinflammatory diseases. Since the brain has a high demand for protein synthesis, it can be vulnerable to disrupting the ER’s function, which causes these different brain diseases. Some of these brain diseases in which ER stress can affect include AD, PD, HD, and ALS.

AD is the most common form of dementia and is a progressive disease that begins with mild memory loss and leads to the loss of the ability to carry on a conversation. ER stress plays a key role in AD as the accumulation of misfolded amyloid-beta and tau proteins, and intracellular calcium homeostasis triggers chronic ER stress [12]. This chronic ER stress results in neuronal dysfunction and cell death, which leads to the decline in memory and speech in a person with AD [12]. Similarly, in PD, a disorder that affects the nervous system, the misfolding and aggregation of a-synuclein proteins cause ER stress and are linked to the degeneration of dopaminergic neurons [13].

ALS is a nervous system disorder that weakens muscles and affects physical function. The accumulation of misfolded proteins, such as SOD1, an antioxidant enzyme that protects against oxidative stress in eukaryotic cells, leads to ER stress. The abnormal aggregation of misfolded proteins triggers a stress response called the ER stress pathway [14]. This ER stress contributes to the loss of neurons and muscle function in humans with ALS. HD is a disorder that causes neurons in the brain to break down and die. An accumulation of misfolded proteins in the ER causes ER stress and activates the UPR mechanisms, which are found to be linked to HD. HD is characterized by mutant Huntington proteins, which causes ER stress and activates apoptotic pathways, which causes the neurons to die [15].

Inflammatory diseases like strokes also induce ER stress. During a stroke, oxygen and nutrients are reduced, which causes an accumulation of misfolded proteins and leads to ER stress and cell death. ER stress plays a role by directly regulating the inflammatory pathways [4]. Chronic ER can have terrible effects on the brain. It leads to cells killing themselves through apoptosis and leaving the brain to die.

**Figure 2.**
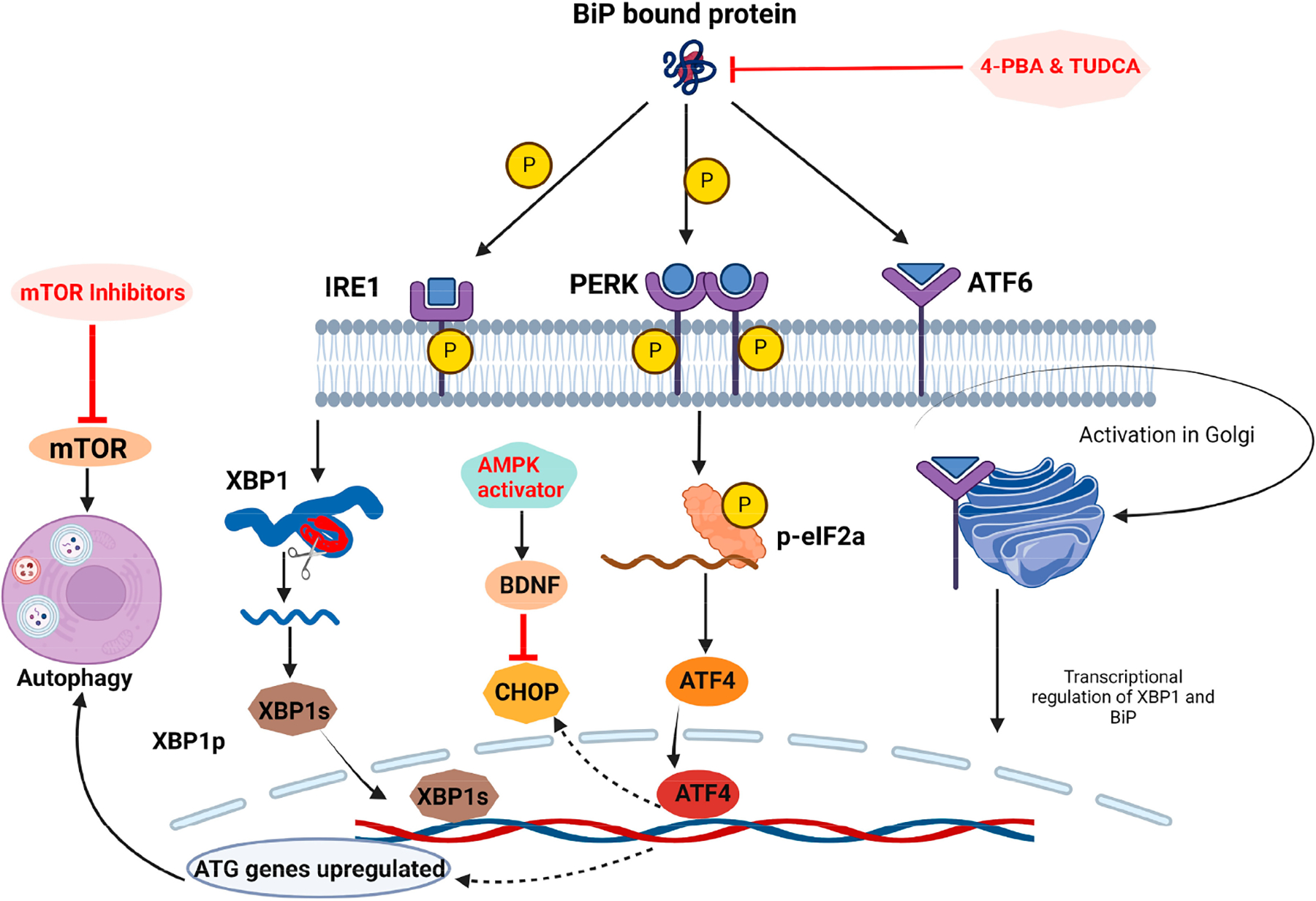
ER stress inhibitor mechanisms. Created in BioRender. Hasan, S. (2024) BioRender.com/h13k351.

Researchers are figuring out ways to alleviate ER stress and reduce its effects. They are attempting to enhance protein folding and lessen the load of misfolded proteins. One of the most direct ways to reduce ER stress is to use chemical chaperones, such as 4-phenylbutyric acid (4-PBA) and tauroursodeoxycholic acid (TUDCA), to facilitate protein folding and to alleviate ER stress [16]. Figure 2 shows ER stress inhibitor mechanisms.

## 4. ER Stress and AD

ER stress plays a significant role in the pathogenesis of AD. The ER is responsible for protein folding and maturation through glycosylation. Protein glycosylation defect causes the accumulation of unfolded or misfolded proteins in the ER, leading to ER stress [17,18]. This disruption triggers a cellular response known as the UPR, which aims to restore normal ER function. However, in chronic conditions such as AD, prolonged ER stress and sustained UPR activation can contribute to disease progression. AD is a severe neurodegenerative disorder and the leading cause of dementia, marked by a gradual decline in cognitive function [19]. The pathology of AD involves the accumulation of amyloid-beta (Aβ) and phosphorylated tau (P-tau) in the brain, with tau being a brain-specific protein that leaks into the plasma in AD. The buildup of tau disrupts ER protein homeostasis, activating the UPR by impairing the ER-associated degradation (ERAD) pathways, which leads to ER stress [20]. In the context of AD, chronic ER stress and dysregulated UPR contribute to the disease progression. Aβ peptides, which are hallmark features of AD, have been shown to induce ER stress by interfering with protein folding and calcium homeostasis within the ER. Persistent ER stress can lead to sustained UPR activation, which, if unresolved, shifts from a protective to a pro-apoptotic response, leading to neuronal death [21]. Furthermore, the hyperphosphorylation of tau, another key pathological feature of AD, is associated with ER stress. Tau pathology exacerbates ER stress, which in turn can promote further tau aggregation and neurodegeneration, creating a vicious cycle [22]. Overall, ER stress and the maladaptive UPR are closely linked to the molecular mechanisms underlying AD, making them potential therapeutic targets for mitigating disease progression.

BiP is the major ER chaperone involved in protein folding, protein maturation, and protein trafficking. It serves as a critical sensor of ER stress, and its expression increases when the unfolded proteins accumulate in the ER lumen. A recent study shows that BiP expression was elevated in an AD mouse model, confirming the presence of ER stress in vivo. In these AD mice, increased levels of IRE1α, p-p38, and p-NF-κB indicate the activation of NF-κB and p38 inflammatory pathways via the IRE1α axis, contributing to neuroinflammation [23]. Mutations in amyloid precursor protein (APP) accelerate the accumulation of toxic Aβ oligomers, leading to ER stress and increasing the GRP78 and p-eIF2α expression [12]. In a mouse model of AD, the deletion of IRE1 reduces APP expression. At the same time, in vitro studies show that inhibiting IRE1 signaling disrupts APP homeostasis, causing APP retention in the ER and inducing ER stress [24]. Interestingly, the overexpression of XBP1 and IRE1 in the nervous system of AD mice has been shown to reduce amyloid deposition and protect synaptic and cognitive functions, suggesting that XBP1 plays a vital role in memory and cognitive functions [25]. The tau protein plays a fundamental role in binding to and stabilizing microtubules in neurons [26]. Abnormal accumulation and aggregation of tau protein in the human brain are hallmark features of tauopathies. In addition to various subcellular abnormalities, tau aggregation in neurons induces ER stress, leading to the activation of the UPR [27]. The hyperphosphorylated tau proteins activate the inflammatory cytokine pathway (NF-kB) by the phosphorylation of PERK [28]. Inflammatory cytokines (interleukin-1β and TNF-α) stimulate the neuronal release of tau and enhance its phosphorylation, facilitating the formation of neurofibrillary tangles that contribute to neurodegenerative alterations [29]. Studies mentioned above targeting ER stress present a promising approach for mitigating tau-mediated neuroinflammation and slowing the progression of tauopathies. The ATF6 gene is pivotal in the stress response of the ER. ATF6, or activating transcription factor 6, is one of the key sensors of ER stress; its expression is elevated when the ER’s capacity to fold proteins properly is overwhelmed, accumulating misfolded proteins [30]. Under normal conditions, ATF6 is located in the ER as an inactive precursor. ATF6 is transported to the Golgi apparatus when ER stress arises and processed into its active form. The active ATF6 then translocates to the nucleus, where it functions as a transcription factor, regulating the expression of genes involved in mitigating ER stress. These genes include those encoding chaperones and other proteins that assist in protein folding and degradation, such as GRP78 (BiP) and XBP1 [31].

**Figure 3.**
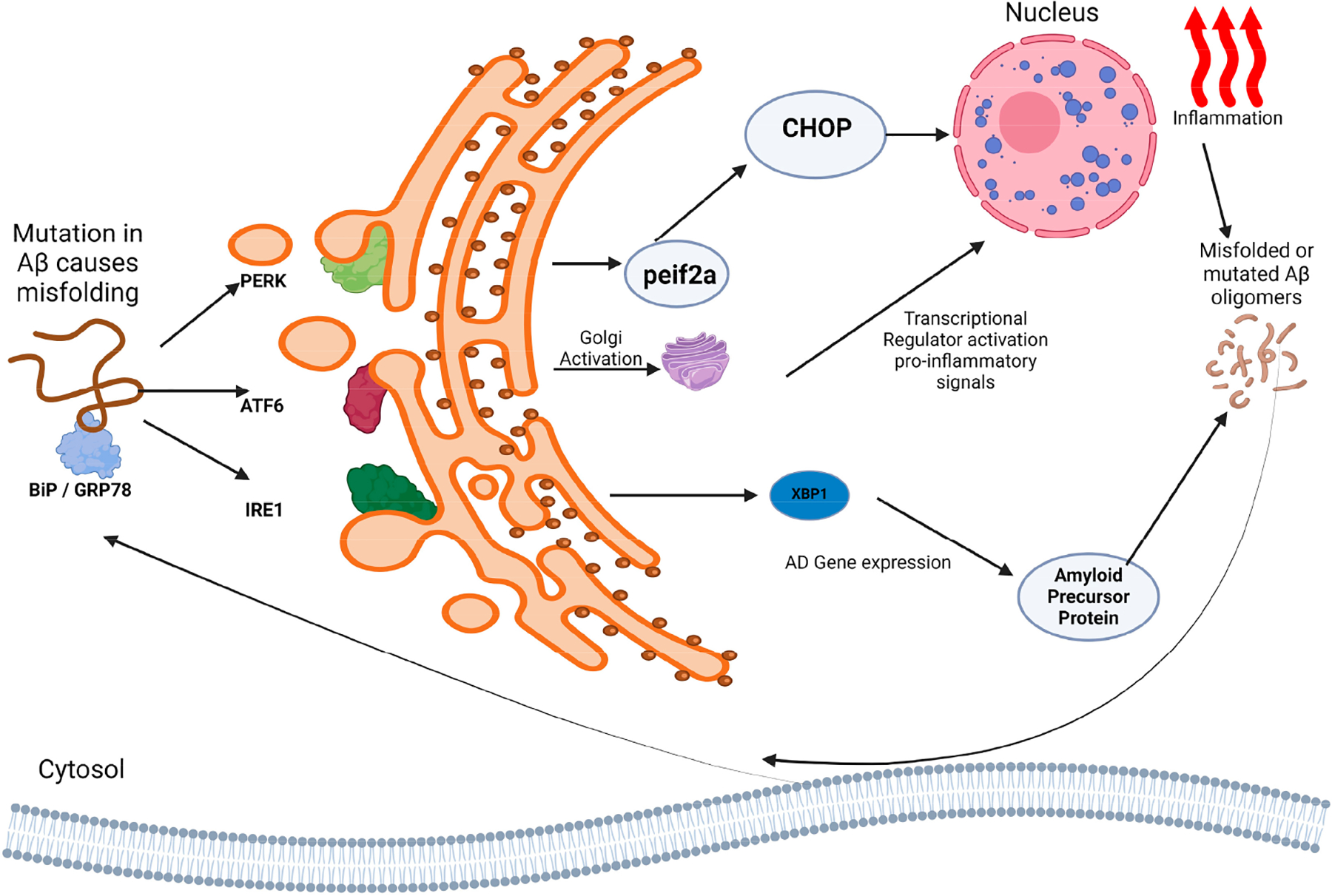
ER Stress and Alzheimer’s disease. Hasan, S. (2024) BioRender.com/p01u830.

The activation of ATF6 is a part of the UPR, a protective mechanism aimed at restoring ER homeostasis. However, persistent ER stress can lead to chronic ATF6 activation, contributing to cellular dysfunction and pathology [32]. In the context of neurodegenerative diseases like AD, the dysregulation of ATF6 has been observed. In AD, a reduced ATF6 expression can impair the cellular stress response, exacerbate the accumulation of Aβ and tau proteins, and contribute to neurodegeneration [33]. This suggests that maintaining proper ATF6 function is crucial for cellular health and mitigating the adverse effects of ER stress in neurodegenerative conditions. Additionally, in an in vitro study using SK-N-SH human neuroblastoma cells, Aβ selectively activates the PERK-eIF2α pathway. Silencing PERK results in cell death, but this effect is countered by Salubrinal, a specific activator of eIF2α [34]. The studies mentioned above suggest that ER stress is predominantly linked with the pathogenesis of AD. Furthermore, the inhibition of ER stress in cellular and animal models has been shown to alleviate AD symptoms, highlighting potential molecular therapeutic targets for effective intervention in AD. Figure 3 shows the cycle involving misfolded protein response and inflammation in AD.

## 5. Clinically Approved ER Stress Inhibitors

Clinically, multiple drugs can modulate ER stress in various cell types and organ systems. Six different drug classes are used to regulate stress in the ER. The first class of drugs is called mTOR inhibitors, which are clinically used to treat various cancers, autoimmune diseases, and neurodegeneration [35]. An example of an mTOR inhibitor is rapamycin, which has been reported to suppress ER stress by activating autophagy pathways to dispose of misfolded proteins properly. While rapamycin is primarily used in the treatment of diabetes, some studies show that rapamycin could be a potential neuroprotective agent for conditions such as traumatic brain injury (TBI) [36,37]. The second class of drugs is called chemical chaperones; these drugs act similarly to endogenous chaperones by displacing and reducing misfolded proteins and promoting mutant protein trafficking [38]. Two examples of these drugs include 4-PBA and TUDCA, which were mentioned previously. 4-PBA and TUDCA have shown highly promising effects in alleviating ER stress in liver hepatocytes, and they effectively mitigate apoptotic promoters such as eif2a, which promote cell survival [39]. 4-PBA has been implied in studies to be neuroprotective against cerebral ischemic conditions, and TUDCA has been shown to be a candidate in slowing the progression of AD [40,41]. The third class of ER modulators is a significant drug class known as the AMPK (adenosine monophosphate-activated protein kinase) activators. AMPK activators are primarily used in overweight or obese patients exhibiting early signs of adipocyte-induced inflammation. Metformin is an excellent example of an AMPK activator, a drug used to treat patients entering the early stages of diabetes mellitus. While metformin is primarily used for diabetes, studies are showing that, like rapamycin, metformin also promotes neuroprotection by upregulating brain-derived neurotrophic factor (BDNF) and has been shown to be effective at restoring locomotor and depressive symptoms in mice [42]. The fourth class of ER modulators is called the glucagon-like peptide receptor (GLP-1) agonists. An example of these drugs is exenatide, which effectively protects the cells from ER stress by upregulating activating transcription factor-4, which helps the cells recover from pro-apoptotic UPR mechanisms [43]. Exenatide was shown to modulate glucose homeostasis in certain brain regions, in a human sample, implying that it could be submitted relatively quickly for an FDA review for certain brain disorders [44]. The fifth class of drugs is called peroxisome proliferator-activated receptor (PPAR) agonists. An example of a PPAR agonist is fenofibrate, a well-known cholesterol medication that has been shown to alleviate ER stress in mouse hepatocytes through the IRE1-XBP1 pathway [41]. Interestingly, fenofibrate has also been studied in the brain and has been shown to help alleviate symptoms of ALS, MS, ischemia-related memory impairment, and TBI [45–47]. The implications of these results are that many different brain disorders are perturbed by ER stress and depend on the brain region, cell type, and underlying cause of the ER stress. The last group of drugs that are used to alleviate ER stress are the angiotensin II type 1 receptor blockers (ARBs). ARBs are widely used to treat hypertension and control blood pressure in patients. An example of an ARB is valsartan, which has been shown to alleviate ER stress by inhibiting the CHOP protein in the PERK pathway in a rat model [48]. By inhibiting CHOP, nuclear translocation cannot occur, and this mitigates the pro-apoptotic signals that the pathway usually regulates. Valsartan has also been shown to have neuroprotective effects, mainly in ischemic conditions, by reducing inflammation and depression/anxiety by promoting the expression of BDNF in the hippocampus [48,49]. Table 1 shows a list of the drugs mentioned as well as their drug classes and the neurological implications that they have.

## 6. Shortcomings of ER Stress Drugs

Despite the promise of targeting ER stress as a therapeutic approach for neurodegenerative diseases and other conditions, there are several notable shortcomings associated with ER stress drugs:

### Specificity and Off-Target Effects:

Many drugs targeting ER stress pathways may not be sufficiently specific, potentially affecting other cellular processes or pathways. For example, broad-spectrum ER stress inhibitors might impact normal cellular functions, leading to unintended side effects or toxicity [48].

### Complexity of ER Stress Pathways:

The ER stress response involves multiple interconnected pathways, including the unfolded protein response (UPR) and ER-associated degradation (ERAD). Drugs that target one aspect of ER stress may inadvertently disrupt the balance of these pathways, leading to suboptimal therapeutic outcomes [49].

### Incomplete Understanding of Mechanisms:

Our understanding of how ER stress contributes to various diseases is still evolving. Incomplete knowledge about the precise mechanisms and interactions within the ER stress response can limit the development of effective and targeted therapies [50].

### Drug Delivery Challenges:

Effective delivery of ER stress-modulating drugs to specific tissues or cells remains a significant challenge, particularly in the central nervous system. Ensuring that drugs reach the target site in appropriate concentrations without causing systemic toxicity is crucial [51].

### Resistance and Adaptation:

Cells may develop resistance to ER stress drugs over time, either by upregulating compensatory stress response mechanisms or through genetic mutations. This can reduce the long-term efficacy of treatments [52].

### Side Effects and Toxicity:

Some ER stress-modulating drugs may adversely disrupt normal protein folding or affect other cellular functions. For instance, the prolonged inhibition of stress response pathways can impair cellular homeostasis and lead to additional complications [2].

### Variability in Patient Response:

Individual variability in genetic and molecular profiles can affect how patients respond to ER stress-targeted therapies. Personalized approaches may be needed to optimize treatment outcomes and minimize adverse effects [53].

While targeting ER stress holds significant potential for therapeutic interventions, addressing these shortcomings is essential to enhance the efficacy and safety of ER stress drugs. Continued research and development are needed to overcome these challenges and achieve the full therapeutic potential of this approach.

## 7. Conclusions

As was shown and discussed, the use of ER stress inhibitors goes much further than the current clinical uses that are being implemented. Many clinical ER stress inhibitors could potentially also be used as a means of neuroprotection; while they are used to treat other conditions such as diabetes, hypertension, and cardiovascular abnormalities, each class of ER stress inhibitor has also shown progressive results in various animal models displaying high neurotrophic and neuroprotective effects. By manipulating the three main UPR pathways (Figure 1), these drugs offer various mechanisms to potentially alleviate ER stress, providing a potential degree of therapeutic diversity. In other words, if each class of medication was approved for the use of neurological diseases, clinicians could use one or more drugs in tandem to potentially treat a severe neurological condition such as ALS, AD, MS, TBI, or even stroke events, both ischemic and hemorrhagic. The key to all the treatments is the reduction in rampant inflammation, which ultimately leads to cell death in the form of apoptosis. It is already well known that various disease conditions cause the misfolding of proteins, which causes the ER protein load to be too much to handle. This leads to the activation of the UPR mechanism and further inflammation. ER stress inhibitors of almost every class offer potential clinical therapy for many different disease conditions that affect the brain. At the same time, challenges are still present in pharmacokinetics and blood–brain barrier penetration, so medicinal chemists and researchers can effectively manipulate each drug’s active site to better formulate an ER stress inhibitor specifically for the brain.

## Figures and Tables

**Table 1.** List of FDA-approved ER stress inhibitors and their implications in neurological conditions.

Drug	Drug Class	Clinical Uses (FDA Approved)	Neurological Implication	Mechanism	Ref.
Rapamycin	mTOR Inhibitor	Cancer Diabetes Kidney Rejection	TBI Protection Anti-Inflammatory	Autophagy Activation	[36,37]
4-PBA and TUDCA	Chemical Chaperones	Urea cycle disorders Biliary Cholangitis	Ischemic Protectant Alzheimer’s Disease	Mitigates apoptotic promoters	[40,41]
Metformin	AMPK Activator	Diabetes	Neuroprotectant	BDNF Expression	[42]
Exenatide	GLP-1 Agonist	Diabetes	Glucose Homeostasis (Human Study)	ATF-4 Upregulation	[44]
Fenofibrate	PPAR Agonist	Dyslipidemia	Multiple Sclerosis ALS Ischemic Memory loss TBI	NF-kβ Suppression Anti-Inflammatory	[45–47]
Valsartan	ARBs	High Blood Pressure Heart Failure	Neuroprotectant	BDNF Expression	[37,38]
